# Sudden Odynophagia and Globus—A Unique Presentation of a Nonsecreting Parathyroid Adenoma: A Case Report and Literature Review

**DOI:** 10.1155/2020/6805805

**Published:** 2020-12-29

**Authors:** Luxman Srikantha, Esmael H. Amjad, Rafic Beydoun

**Affiliations:** ^1^Department of Otolaryngology-Head and Neck Surgery, Detroit Medical Center, Detroit, MI, USA; ^2^Department of Pathology, Detroit Medical Center, Detroit, MI, USA

## Abstract

Parathyroid adenomas are most commonly diagnosed when symptoms consistent with primary hyperparathyroidism arise. However, certain parathyroid glands may enlarge without such symptoms. Described here is a case in which a patient presented with acute signs of unilateral cervical point tenderness, dysphagia, and odynophagia. Calcium and parathyroid hormone levels tested within normal range. Imaging revealed an enlarged right-sided mass, with compression of the trachea-esophageal groove and potentially the right recurrent laryngeal nerve. Surgical excision was performed, and final pathology revealed an infarcted parathyroid adenoma. Clinical symptoms promptly resolved thereafter. Current NIH criteria for parathyroidectomy include various symptoms of hyperparathyroidism but do not include the above findings. Nonsecreting parathyroid adenomas rarely cause laryngeal symptoms, as this has only been documented once before.

## 1. Introduction

Parathyroid adenomas (PAs) are by far the most common cause of primary hyperparathyroidism. The pathogenesis of their development is thought to arise from clonal expansion caused by genetic mutation [1]. Altered sensitivity to calcium may contribute to their hypersecretion of parathyroid hormone (PTH). Consequently, most of the symptoms associated with PAs are those of hyperparathyroidism (HPT): fatigue, constipation, myalgias, and muscle weakness [2].

Here, we discuss a unique case in which a patient presented to our institution with acute unilateral cervical tenderness, dysphagia, odynophagia, globus pharyngeus, and dysphonia as her only symptoms. Lab values, including PTH and calcium, were within normal limits. Upon evaluation, she was found to have a mass posterior to the right thyroid lobe. Pathological examination revealed an infarcted parathyroid adenoma. There has been only one such case previously reported in the literature [3].

## 2. Case Report

A 42-year-old African American female presented to the emergency room with concerns of acute neck pain, dysphagia, odynophagia, globus pharyngeus, and dysphonia that started three days prior. Her symptoms progressively worsened over this time; she sought medical care chiefly for reduced cervical range of motion and strangled voice. She was able to localize the pain to her right anterior neck. She denied any previous episodes or alleviating/aggravating factors. The patient noted being under the care of an endocrinologist as she had a history of hyperthyroidism. She was currently euthyroid on 5 mg of methimazole. She had no history of elevated PTH or symptoms associated with hypercalcemia. Other contributory history included sick-cell trait and a tobacco abuse history of one-half packs per day for 28 years.

**Figure 1:**
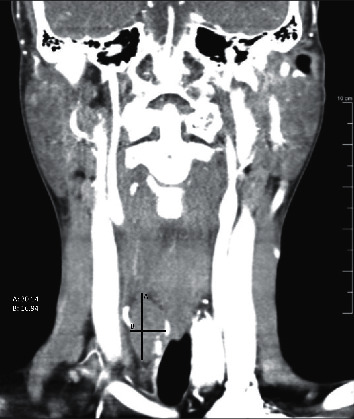
Coronal CT-neck with contrast demonstrates a 30.1 mm × 16.9 mm right-sided parathyroid adenoma causing mass effect.

**Figure 2:**
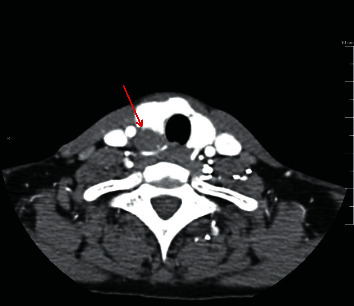
Axial CT-neck with contrast reveals an enlarged right inferior parathyroid gland (*red arrow*).

A CT-neck with contrast was obtained as part of her work-up, which revealed a 3.1 × 1.2 × 1.7 cm solid mass posterior to the right thyroid lobe (Figures 1 and 2). The lesion itself showed hypoattenuation when compared to the adjacent thyroid, but this was likely due to the contrast dye being entirely in the arterial phase at the time of imaging. The lesion was noted near the tracheoesophageal groove. The patient did have a previous thyroid ultrasound two years prior, which showed a diffusely enlarged thyroid. No parathyroid enlargement or other abnormality was mentioned at that time.

The patient was admitted for pain control and for work-up of the newly diagnosed neck mass. A core-needle biopsy was obtained which grossly appeared dark red-black and serosanguineous. Histopathologic evaluation of the specimen revealed small foci of endocrine glandular tissue, which lacked follicular or colloid architecture, thus ruling out thyroid origin. Immunohistochemistry for parathyroid hormone performed on the cellblock was negative; however, the overall features of the specimen were consistent with parathyroid tissue. Features of malignancy were not seen. After the biopsy was obtained, the patient's pain slightly improved, but voice was still strangled. A flexible fiberoptic laryngoscopy was performed by the authors, which revealed no laryngeal pathology, dysfunction, or airway compromise.

Due to the patient's concerning symptoms associated with her neck mass, she was scheduled for surgery. Two days prior to surgery, her labs were as follows: PTH—68 pg/mL (12–88), total T3—84 ng/dL (87–178), free T4—0.6 ng/dL (0.7–1.7), TSH—1.44 *μ*IU/mL (0.45–5.33), and calcium—9.1 mg/dL (8.6–10.8). The operation was uneventful. The mass was encountered posterior to the right thyroid gland inferiorly. Fibrotic adhesions to the thyroid gland as well as the tracheoesophageal groove were noted during dissection. Grossly, the mass was oval, solid (not cystic), soft, and yellow-brown in color. It did not have a fibrofatty appearance. Intraoperatively, the right recurrent laryngeal nerve was identified in close approximation to the mass.

The patient was kept under observation for pain control and management of the indwelling Jackson–Pratt drain. Postoperatively, her symptoms markedly improved. Her voice was intelligible without strangulation. Additionally, she admitted oral dietary tolerance without symptoms of dysphagia or pain.

**Figure 3:**
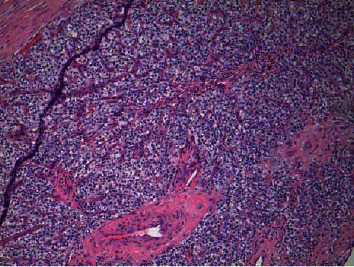
A hematoxylin and eosin stain clearly showing a well-defined rim of encapsulated hypercellular parathyroid tissue composed of chief cells and oxyphil cells.

Grossly, the mass was described as a well-encapsulated dark pink-red soft tissue mass weighing 0.98 grams. Upon sectioning, a 1.8 × 0.8 × 0.6 cm well-circumscribed variegated tan-yellow to dark red focally softened nodule was present with all slices coming to within <0.1 cm of all margins. A rim of scant pink-tan grossly uninvolved soft tissue was located at the periphery. Microscopical sections revealed a well-demarcated nodule with encapsulated, cellular, homogenous cells composed of chief cells with some oxyphil cells in a delicate capillary network (Figure 3). There was a defined rim of normal parathyroid tissue with mostly central necrosis and thrombosed vessels. Immunohistochemical stain for parathyroid hormone was positive confirming the parathyroid nature of the mass. The patient's PTH did decrease to 26 pg/mL, and calcium remained within normal limits measured at 8.7 mg/dL.

## 3. Discussion

PAs are the most common cause of primary hyperparathyroidism. The prevalence of primary hyperparathyroidism is 21 cases per 100,000 person-years, and this most likely is an underdiagnosed condition [4]. There are many instances in the literature which identify unique presentations of parathyroid enlargement, including adenomas, hyperplasia, cysts, and carcinomas. Additionally, there are many clinical scenarios that present with dysphonia and cervical pain including benign and malignant laryngeal tumors, invasive thyroid tumors, vagal schwannomas, cervical paragangliomas, and parapharyngeal space tumors. These patients with adenomas generally present in a typical fashion. They start to experience symptoms of fatigue, constipation, muscle and bone pain, dyspepsia, and mental changes; they may have also been diagnosed with nephrolithiasis, renal dysfunction, osteopenia, or gastric ulcers. After undergoing routine labs, high levels of calcium would generally prompt a clinician to investigate intact-PTH levels, and a diagnosis would be confirmed with additional imaging or biopsies.

The incidence of nonfunctioning PAs is exceeding low. Whether this is due to a rare distinct genetic mutation, a rare inactivation event affecting PTH production or secretion in a previously functioning PA, or simply due to relative clinical silence is unclear [5]. However, this is not the first time a nonsecreting PA has been described. Although published in 2001, Poppe et al. described a 31-year-old male that presented in 1990 with a firm mass in the left cervical region with symptoms of pressure for 2 months [6]. Removed via surgical excision, histology confirmed PA while all relative serum lab values remained normal. Again in 2001, Marchesi et al. published a retrospective study of 1400 thyroidectomy patients over a 13-year period [7]. They noted that 3 patients who had parathyroid enlargement had preoperative PTH levels within normal limits. Due to the possibility of a nonfunctioning adenoma developing into one that is functioning, they recommended routine removal of these “incidentalomas” if performing thyroid surgery.

Nonfunctioning parathyroid carcinomas (PCs) have also been described, whereas this entity usually presents with high levels of PTH; when nonfunctioning, they are more aggressive and portend a poorer prognosis [8]. Due to their relatively silent clinical presentation, they likely will only present when larger than their functioning counterparts, resulting in a neck mass with possible dysphonia and/or dysphagia as the presenting symptoms.

In our case, serum intact-PTH was not elevated. Immunohistochemical staining demonstrated PTH within the adenoma. The most likely explanation for this clinical picture is that the infarction evidenced on pathologic evaluation caused a decrease in secretion of PTH into the bloodstream.

The description of vocal fold dysfunction or acute dysphonia from parathyroid pathology is not novel. In as early as 1972, Lore Jr. et al. had described PAs causing vocal fold paralysis [9]. In 1990, Ben-Shlomo et al. reported a case of sudden dysphonia due to parathyroid apoplexy [10]. The patient was a 59-year-old male that also presented with acute hoarseness and a palpable mass. Indirect laryngoscopy revealed right vocal fold paralysis, and at the time of surgery, they encountered a dark, blood filled well-encapsulated mass posterior to the right inferior thyroid. Histology confirmed the growth as an acute hemorrhage of a hyperplastic parathyroid gland, distinctly different from adenoma. In 1999, another case of parathyroid apoplexy was described to cause sudden recurrent laryngeal nerve paralysis [11]. The patient was a 64-year-old male that presented with acute onset hoarseness and right neck pain. Physical exam revealed vocal fold paralysis. Laboratory testing revealed hypercalcemia and an elevated PTH of 120 pg/mL (15–50). Needle aspiration revealed brown-fluid, and a right PA was ultimately resected. More recently, Lee and colleagues described a 2.0 × 2.5 cm PA causing hoarseness and dysphagia for 1 month [12]. Flexible laryngoscopy revealed vocal fold paralysis. Serum intact-PTH levels were markedly elevated.

Several causes of vocal fold paralysis from PAs have been described. Rapid expansion from spontaneous hemorrhage has been thought to apply pressure to the adjacent recurrent laryngeal nerve in some cases. In other cases, the sheer size of a large solid PA can cause a mass effect. The resulting stretching and pressure from either of these scenarios can cause neuropraxia or axonotmesis and subsequent impaired nerve conduction [13]. One must also be concerned about the possibility of PC, where direct involvement of the nerve causes dysfunction.

In our case, due to the acuity of the dysphonia and associated pain and in correlation with the final pathology, we do believe a recent hemorrhagic event occurred within the adenoma. This has been known to happen in the absence of a causative trauma. True vocal fold palsy may have been averted in our case by the promptness of her presentation (only three days after symptom onset), needle aspiration (decompression of the recurrent laryngeal nerve), and timely surgical excision.

The unique sequela of vocal fold dysfunction due to a nonfunctioning PA has only been described once before. Recently, in 2016, Kamali et al. described a 49-year-old male with a 10-week history of dysphonia and globus pharyngeus [3]. Interestingly, this patient did have a history of nephrolithiasis but was never diagnosed with HPT. Flexible nasal endoscopy demonstrated paralysis of the left vocal fold, and CT imaging revealed a cystic lesion about 1.8 cm adjacent to the esophagus. Serum PTH, calcium, thyroid-stimulating hormone, free thyroxine, and free triiodothyronine were all within their institution's normal range.

The management of parathyroid glands has been studied and, over time, adjusted. For the most part, the criteria for the surgical excision of parathyroid glands, initially proposed in 1990 by the NIH (Table 1) [14], focus on the effects of HPT. These criteria attempt to measure the detrimental effects of clinically asymptomatic HPT on the patient, guiding the clinician on when these effects are severe enough to recommend surgical intervention. In contrast, symptoms of HPT including fractures, nephrolithiasis, neuromuscular symptoms, and psychosis are clearly severe and obviate surgical excision.

Unrecognized in much of the literature are the symptoms of a neck mass with cervical tenderness, dysphagia, and dysphonia associated with a parathyroid growth. These would be the only presenting symptoms of a nonfunctioning parathyroid growth and indeed some functioning growths in asymptomatic HPT patients. We believe these masses should be excised, and as such, we propose that the symptoms of cervical tenderness, dysphagia, and dysphonia be added to the guidelines for completeness.

This case report is not immune to limitations. Fiberoptic laryngoscopy was performed only after needle aspiration due to scope availability. The needle aspiration may have decompressed the recurrent laryngeal nerve allowing for return of function. We assume the infarction event was acute and likely causal to the PAs nonfunction, yet the intact-PTH level drawn prior to any symptoms was within normal limits. This lab draw may predate the presence of the PA, as there was no evidence of PA on an ultrasound performed around the same time as the lab draw. Additionally, elevated serum calcium was noted in that same routine endocrine lab draw. Although the patient denied any hematuria or flank pain and creatinine clearance showed no decay of renal function, no urine calcium was on refold to disprove familial hypocalciuric hypercalcemia.

## 4. Conclusion

In conclusion, there have been many instances in the literature that have well described parathyroid enlargement and its proximal effect on the recurrent laryngeal nerve. However, in the absence of hyperparathyroidism, a diagnosis of a PA is highly unlikely. Our patient presented with acute neck pain, dysphonia, and odynophagia, which ultimately was caused by a nonfunctioning PA. It is only the second time in literature that this has been described. Although not addressed in the current guidelines as indications for removal, we recommend that parathyroid glands presenting with the symptoms of neck mass with cervical tenderness, dysphagia, and dysphonia be surgically removed.

## Figures and Tables

**Table 1:** Comparison of guidelines for surgery of asymptomatic HPT patients.

	1990	2002	2008	2013
Serum calcium greater than upper limit of normal	1–1.6 mg/dL	1 mg/dL	1 mg/dL	1 mg/dL
Skeletal	BMD by DXA: *Z*-score less than −2.0 (site unspecified)	BMD by DXA: *T*-score less than −2.5 at any site	BMD by DXA: *T*-score less than −2.5 at any site; previous fragility fracture	BMD by DXA: *T*-score less than −2.5 at lumbar spine, total hip, femoral neck, or distal 2/3 radius vertebral fracture by X-ray, CT, MRI, or VFA
Renal	eGFR reduced by greater than 30% from expected 24 h urine calcium greater than 400 mg/d	eGFR reduced by greater than 30% from expected 24 h urine calcium greater than 400 mg/d	eGFR less than 60 cc/min 24 h urine for calcium not recommended	Creatinine clearance less than 60 cc/min 24 h urine >400 mg/day and increased stone risk by biochemical stone risk analysis; presence of nephrolithiasis or nephrocalcinosis by X-ray, ultrasound, or CT
Age (years)	<50	<50	<50	<50
